# NeighborNet: improved algorithms and implementation

**DOI:** 10.3389/fbinf.2023.1178600

**Published:** 2023-09-20

**Authors:** David Bryant, Daniel H. Huson

**Affiliations:** ^1^ Department of Mathematics and Statistics, University of Otago, Dunedin, New Zealand; ^2^ Algorithms in Bioinformatics, University of Tübingen, Tübingen, Germany; ^3^ Cluster of Excellence: Controlling Microbes to Fight Infection, University of Tübingen, Tübingen, Germany

**Keywords:** NeighborNet, phylogenetic networks, SplitsTree, split networks, planar graph drawing

## Abstract

NeighborNet constructs phylogenetic networks to visualize distance data. It is a popular method used in a wide range of applications. While several studies have investigated its mathematical features, here we focus on computational aspects. The algorithm operates in three steps. We present a new simplified formulation of the first step, which aims at computing a circular ordering. We provide the first technical description of the second step, the estimation of split weights. We review the third step by constructing and drawing the network. Finally, we discuss how the networks might best be interpreted, review related approaches, and present some open questions.

## 1 Introduction

**FIGURE 1:**
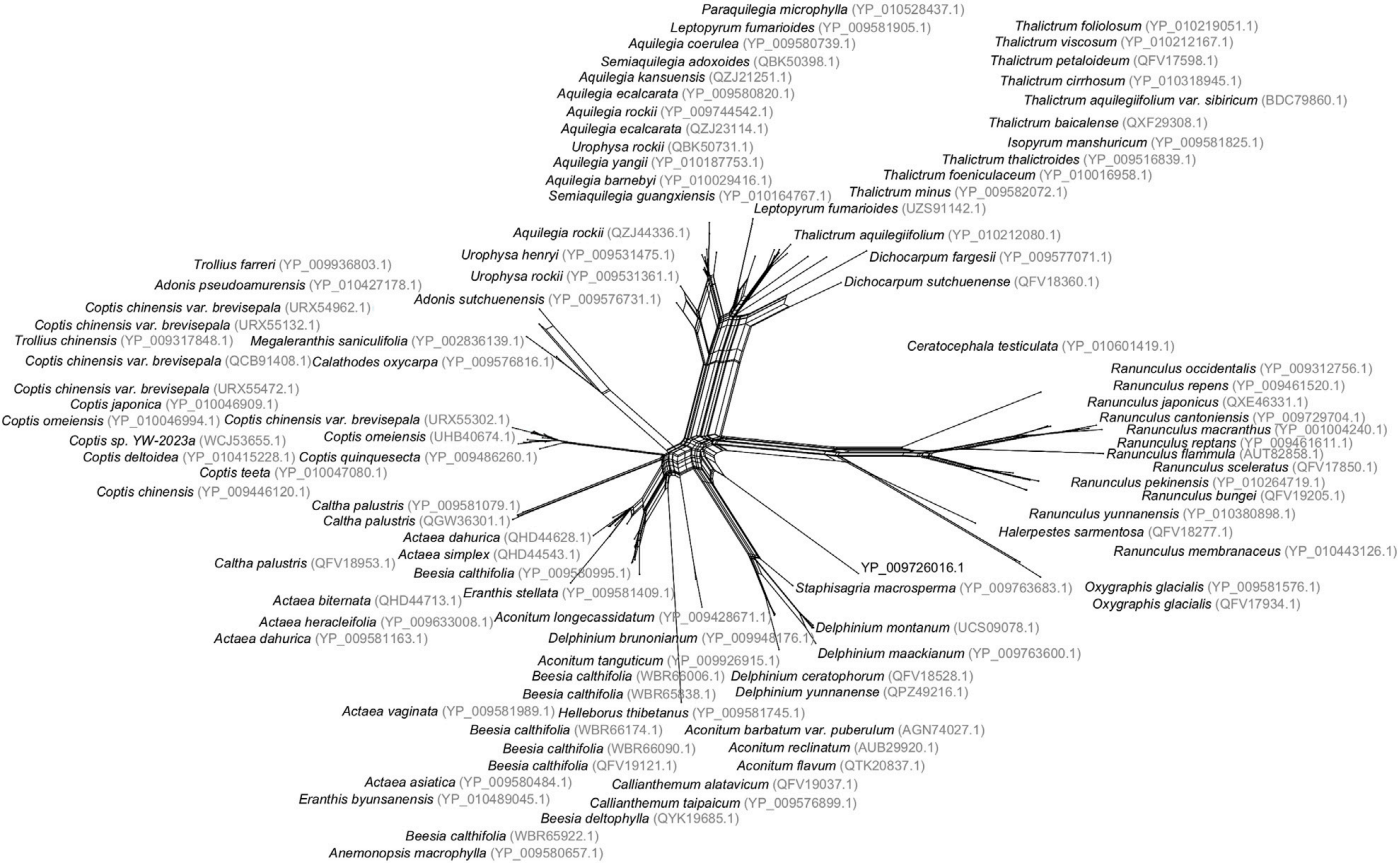
Split network of 100 buttercup and related species, computed using the NeighborNet method, based on distances inferred from a multiple sequence alignment of protein sequences of the *cytochrome c* gene, downloaded from NCBI. This analysis is available in the examples directory of SplitsTreeCE.

Evolutionary relationships between species are usually visualized using a phylogenetic tree. When reticulate events are suspected to play an important role, a phylogenetic network is sometimes considered a more suitable representation. Even when reticulation is not present, networks can be useful for detecting problems with the data or ambiguities in the inferred phylogeny. One of the most widely used methods for computing such networks is NeighborNet, which was published over 20 years ago (Bryant and Moulton, 2002; Bryant and Moulton, 2004). It takes a distance matrix as input and produces a planar network as output, aiming to show both evolutionary relationships and conflicts in the data (see Figure 1).

The NeighborNet method has been applied within a wide range of contexts. A cursory survey of recent citations reveals applications to sea slugs, monkey pox, angelica, daisies, butterfly parasites, linguistics, entomopathogenic fungi, mussels, and Wenchang chickens. The main appeal of the method is that it does not force the given data onto a single phylogenetic tree, but instead can display incompatibilities in the data.

The computation of a phylogenetic network using the NeighborNet is performed in three main steps:

1. An agglomerative algorithm is used to compute a circular ordering of the taxa.
2. Non-negative least squares (NNLS) are used to estimate the split weights compatible with the given ordering.
3. A split network construction method is used to calculate the final network.

In this article, we first provide a new, simplified description of the agglomerative algorithm, focusing directly on the task of computing a cycle. We then describe (for the first time) the best-performing methods used to estimate split weights. This includes novel, low-level matrix multiplication algorithms and a recent survey of relevant NNLS optimization algorithms, followed by a review of techniques for constructing the network from a set of splits. We provide some thoughts on the interpretation and misinterpretation of NeighborNet and conclude with a list of open questions.

## 2 Splits, compatibility, and circularity

Throughout this article, we use *X* to denote a set of taxa of size *n*. We use S to denote a set of splits on *X*, where any split *S* = *A*|*B* consists of two non-empty, disjoint subsets *A* and *B* whose union equals *X*. Usually, a non-negative weight *λ*(*S*) is associated with each split *S*. A split is called trivial if one of its two parts has cardinality 1. For any split *S* = *A*|*B*, let *S*(*x*) and $\bar{S}\left(x\right)$ denote the split part that contains *x* or that does not contain *x*, respectively.

For a set of splits S with weights *λ*, the split distance between two taxa *x* and *y* is defined as

$$d_{S}\left(x,y\right)=\underset{\begin{array}{c} S\in S:\\ S\left(x\right)\neq S\left(y\right) \end{array}}{\sum }\lambda \left(S\right),$$

where the sum is over all splits that separate *x* and *y*, that is, contain *x* and *y* in separate parts.

Let *T* be a phylogenetic tree on *X*, that is, a tree with no nodes of degree 2 and leaves labeled by elements of *X* one-to-one. Any edge (or branch) *e* in *T* defines a unique split *S* = *A*|*B* on *X*, with *A* and *B* being the set of taxa reachable from one end of *e* or the other, respectively, without crossing *e*. The set S(T) of all splits associated with *T* is called the split encoding of *T*, and a classic result states that any given set of splits S on *X* is the split encoding of some phylogenetic tree *T* if, and only if, S is “compatible” and contains all trivial splits (Buneman, 1971).

A distance matrix *d* on *X* is called *additive* if there exists a phylogenetic tree *T* such that, for any two taxa *x* and *y*, the sum of edge weights along the path that connects them in *T* equals *d*(*x*, *y*). Equivalently, formulated in terms of splits, *d* is additive if and only if there exists a compatible set of splits S on *X* and weights such that $d=d_{S}$ .

A set of splits S on *X* is called *circular* if there exists an ordering *θ* = (*x*_1_, *x*_2_, *…*, *x*_*n*_) of the set of taxa such that, for each split S∈S , the elements of $\bar{S}\left(x_{1}\right)$ , with the split part that does not contain *x*_1_, appear consecutively in the ordering (Bandelt and Dress, 1992b). This property is of interest because any circular set of splits can be clearly visualized by a planar network with all the taxa appearing on the perimeter of the network (Dress and Huson, 2004). The circular ordering determines in which order the taxa are encountered as one circumnavigates the network.

A distance matrix *d* is called *circular* if there exists a *circular* set of splits S such that $d=d_{S}$ .

## 3 First step: calculation of a circular ordering

Linkage cluster algorithms (Johnson, 1967) and distance-based phylogenetic tree algorithms such as the neighbor-joining algorithm (Saitou and Nei, 1987) use an agglomerative approach to constructing a tree. Initially, all *n* taxa are placed on isolated nodes. The methods then choose two connected components of the graph and link them via a new parent node. This is repeated until the graph is connected, and the result is a tree. Algorithms differ by how they select which components to link and how they set the edge lengths.

Here, we use an agglomerative approach to create a *cycle* rather than a tree. The cycle defines a circular ordering *θ* of the taxa. The general outline of the method is presented in Algorithm 1, where we use *G* = (*V*, *E*) to denote a graph with node set *V* and edge set *E*.

Algorithm 1 NeighborNet: Agglomerative Cycle Calculation

1: set *G* ← (*X*, ∅) ⊳ Initially, *n* isolated nodes
2: set *A* ← *X* ⊳ set of “active” nodes
3: **while** *G* has ≥2 connected components **do**
4:  *select* two different connected components *P* and *Q* in *G*, with |*P* ∩ *A*| ≤ |*Q* ∩ *A*|
5:  **if** *P* and *Q* are both singletons **then**
6:   we have *P* = {*p*} and *Q* = {*q*} with *p*, *q* ∈ *A*
7:   create a new edge (*p*, *q*) ∈ *E*
8:  **else** **if** *P* is a singleton and *Q* is a chain **then**
9:   we have *P* = {*p*} with *p* ∈ *A* and |*Q* ∩ *A*| = 2
10:   *select* *q* ∈ *Q* ∩ *A*
11:   create a new edge (*p*, *q*) ∈ *E* and remove *q* from *A*
12:  **else**⊳ *P* and *Q* both chains
13:   we have |*P* ∩ *A*| = 2 and |*Q* ∩ *A*| = 2
14:   *select* *p* ∈ *P* ∩ *A* and *q* ∈ *Q* ∩ *A*
15:   create a new edge (*p*, *q*) ∈ *E* and remove *p*, *q* from *A*
16:  **end** **if**
17: **end** **while**
18: create a new edge (*p*, *q*) ∈ *E* between the two remaining active nodes *p*, *q* ∈ *A*
19: **return** graph *G* = (*X*, *E*), consisting of a single cycle

Proposition 1 *Algorithm *1 *returns a cycle*.

To see that Proposition 1 holds, note that if the first conditional expression is true, then two isolated nodes are connected by an edge, forming a chain of length 1 between two active nodes. If the second condition holds, then a chain *Q* is extended by a node *p* and the set of active nodes is updated to ensure that precisely the two ends of the extended chain are active. Otherwise, two chains *P* and *Q* are concatenated into a single chain and the set of active nodes is updated to ensure that the chain contains precisely two active nodes, one at each end. Each iteration reduces the number of connected components by one and so the **while** loop will terminate after *n* − 1 iterations.

Assume that we are given a distance matrix *d* on *X* as input. To complete the definition of the agglomerative part of the NeighborNet, we have to specify how the three different selections are respectively made in lines 4, 10, and 14 on the basis of *d*. Throughout Algorithm 1, we maintain and update the matrix *d* on *A*, the set of active nodes or taxa.

For any two connected components *P* and *Q*, we define the distance between *P* and *Q* as the average distance between the active nodes contained in *P* and the active nodes contained in *Q*, that is,

$$d\left(P,Q\right)=\frac{1}{\left|P\cap A\|Q\cap A\right|}\underset{p\in P\cap A}{\sum }\underset{q\in Q\cap A}{\sum }d\left(p,q\right).$$

In line 4, we select a pair of components *P* and *Q* that minimizes the adjusted distance

$$d^{\prime }\left(P,Q\right)=\left(m-2\right)d\left(P,Q\right)-\underset{S}{\sum }d\left(P,S\right)-\underset{S}{\sum }d\left(Q,S\right),$$

summing over all components *S* ≠ *P*, *Q*, with *m* as the total number of components.

In line 10, we select the node *q* = *Q* ∩ *A* for which the adjusted distance between *p* and *q* is minimized. In more detail, for *q* ∈ {*q*_1_, *q*_2_} = *Q* ∩ *A*, we define

$$\begin{array}{cc} r\left(p\right) & =d\left(q_{1},p\right)+d\left(q_{2},p\right)+\underset{S}{\sum }d^{\prime }\left(\left\{p\right\},S\right),\\ r\left(q\right) & =d\left(q_{1},q_{2}\right)+d\left(q,p\right)+\underset{S}{\sum }d^{\prime }\left(\left\{q\right\},S\right),\\ d^{\prime }\left(q,p\right) & =\left(m-1\right)d\left(q,p\right)-r\left(q\right)-r\left(p\right), \end{array}$$

summing over all components *S* ≠ *P*, *Q*. We select *q* = *q*_1_ if *d*′(*q*_1_, *p*) ≤ *d*′(*q*_2_, *p*), else select *q* = *q*_2_.

Similarly, in line 14 we select the pair of nodes *p* and *q* that have minimal adjusted distance. In more detail, for *p* ∈ {*p*_1_, *p*_2_} = *P* ∩ *A* and *q* ∈ {*q*_1_, *q*_2_} = *Q* ∩ *A*, we define

$$\begin{array}{cc} r\left(p\right) & =\frac{1}{2}\left(d\left(p,q_{1}\right)+d\left(p,q_{2}\right)\right)+\underset{S}{\sum }d^{\prime }\left(\left\{p\right\},S\right),\\ r\left(q\right) & =\frac{1}{2}\left(d\left(p_{1},q\right)+d\left(p_{2},q\right)\right)+\underset{S}{\sum }d^{\prime }\left(\left\{q\right\},S\right),\\ d^{\prime }\left(p,q\right) & =m\;d\left(p,q\right)-r\left(p\right)-r\left(q\right), \end{array}$$

summing over all components *S* ≠ *P*, *Q*. We select *p* and *q* that minimize *d*′(*p*, *q*).

We now describe how to update *d*. In the first conditional statement, the set of active nodes *A* is not changed and so *d* does not require updating.

In the second conditional statement, the selected node *q* is removed from the active set *A* and we update

$$d\left(p,\bar{q}\right)\leftarrow \frac{1}{3}\left(d\left(p,\bar{q}\right)+d\left(\bar{q},q\right)+d\left(p,q\right)\right),$$

where $\bar{q}$ denotes the node *not* selected from {*q*_1_, *q*_2_} = *Q* ∩ *A*. For all other active nodes $r\;\left(\neq p,\bar{q}\right)$ , we set

$$d\left(p,r\right)\leftarrow \frac{2}{3}d\left(p,r\right)+\frac{1}{3}d\left(q,r\right)\;\text{and}\;d\left(\bar{q},r\right)\leftarrow \frac{2}{3}d\left(\bar{q},r\right)+\frac{1}{3}d\left(q,r\right).$$

In the third conditional statement, the selected nodes *p* and *q* are removed from the active set *A* and we update

$$d\left(\bar{p},\bar{q}\right)\leftarrow \frac{1}{6}\left(d\left(\bar{p},p\right)+d\left(\bar{p},q\right)+d\left(\bar{p},\bar{q}\right)+d\left(p,q\right)+d\left(p,\bar{q}\right)+d\left(q,\bar{q}\right)\right),$$

where $\bar{p}$ and $\bar{q}$ denote the two nodes that were *not* selected. For all other active nodes $r\;\left(\neq \bar{p},\bar{q}\right)$ , we set

$$d\left(\bar{p},r\right)\leftarrow \frac{1}{2}d\left(\bar{p},r\right)+\frac{1}{3}d\left(p,r\right)+\frac{1}{6}d\left(q,r\right)\;\text{and}\;d\left(\bar{q},r\right)\leftarrow \frac{1}{6}d\left(p,r\right)+\frac{1}{3}d\left(q,r\right)+\frac{1}{2}d\left(\bar{q},r\right).$$ (1)

In previous descriptions of the algorithm, the update was performed by applying the update formulas of the second conditional statement twice, first to $\bar{p}$ and $\left\{q,\bar{q}\right\}$ , and then to $\bar{q}$ and $\left\{p,\bar{p}\right\}$ , potentially introducing an order dependency (Guo and Grünewald, 2023). The new formula (1) fixes this shortcoming, although we note that it can return a different circular ordering than the earlier algorithm in some cases.

These calculations for selecting components and nodes, and for updating *d*, may seem quite complicated; however, they ensure that, if the input distance matrix is circular, then the computed circular ordering belongs to the associated set of circular splits. This is based on the following result.

Theorem 2 (Bryant et al., 2007; Levy and Pachter, 2011). *Let* *d* *be a circular metric on* *X* *and let* *n* = |*X*|*. The pair* *x*, *y* *minimizing*

$$d^{\prime }\left(x,y\right)=\left(n-2\right)d\left(x,y\right)-\underset{z}{\sum }d\left(x,z\right)-\underset{z}{\sum }d\left(y,z\right),$$ (2)

*is adjacent in some circular ordering compatible with* *d* *.*

Note that Eq. 2 is the criterion used to select components to agglomerate in the neighbor-joining algorithm. If the input distances *d* are additive, then the pair *x*, *y* minimizing *d*′(*x*, *y*) corresponds to a cherry (leaves adjacent to the same internal node); for a simple proof of this fact, see Bryant (2005). It is remarkable that this result extends to circular metrics.

In the next section, we will discuss how to compute a set of weighted splits that are compatible with the calculated ordering, and we have the following result (Bryant et al., 2007).

Theorem 3 *NeighborNet is consistent on circular distance matrices. In more detail, let* *d* *be a distance matrix on* *X* *and let* S *be the set of splits computed by steps 1 and 2 of the NeighborNet algorithm. Then*, *we have* $d=d_{S}$ *if and only if* *d* *is circular.*

## 4 Second step: estimation of split weights

The first step of the NeighborNet method computes a circular ordering *θ*. We now describe the second step, in which we set up all splits that are compatible with the given ordering and then use least squares to determine their weights. This is a difficult problem to tackle in practice, and we discuss multiple algorithms to address it.

### 4.1 Setting up the problem

#### 4.1.1 Linear algebra

Suppose we have a distance matrix *d* and a circular ordering *θ* = (*x*_1_, *x*_2_, …, *x*_*n*_) of the taxa. There is a set of *O*(*n* ^2^) splits that are compatible with any such ordering, given by

$$S=\left\{\left\{x_{p},\ldots ,x_{q}\right\}|X-\left\{x_{p},\ldots x_{q}\right\}:1<p\leq q\leq n\right\}.$$

Any choice of non-negative weights $\left\{\lambda_{A|B}:A|B\in S\right\}$ for those splits gives rise to a circular metric $\hat{d}$ via

$$\hat{d}=\underset{A|B\in S}{\sum }{\hat{\lambda }}_{A|B}\delta_{A|B},$$

where *δ*_*A*|*B*_ denotes the semi-metric defined as

$$\delta_{A|B}\left(i,j\right)=\left\{\begin{array}{cc} 1\; & \text{ if }A|B\text{ separates }i\text{ and }j,\\ 0\; & \text{ otherwise.} \end{array}\right.$$

Note that $\hat{d}$ is the circular network analog of the additive distances given by a tree. The aim is to select the split weights so that this inferred metric $\hat{d}$ is as close as possible to the observed distances *d*. Specifically, we aim to choose non-negative weights $\left\{\lambda_{A|B}:A|B\in S\right\}$ that minimize the sum

$$\underset{i,j}{\sum }{\left(d\left(i,j\right)-\hat{d}\left(i,j\right)\right)}^{2}.$$

This is an example of a NNLS problem.

The first step is to rewrite the problem using linear algebra.

Definition 4 *Let* *θ* = (*x*_1_, *x*_2_, …, *x*_*n*_) *be an ordering of the taxa* *X* *. For each* *k* < *ℓ* *, let* *σ*_(*kℓ*)_ *denote the split* $\left\{x_{k},\ldots ,x_{\ell -1}\right\}|\bar{\left\{x_{k},\ldots ,x_{\ell -1}\right\}}$ *so that*

$$S=\left\{\sigma_{k\ell }:1\leq k<\ell \leq n\right\}.$$

*Let* **A** *denote the* n2×n2 *matrix with rows indexed by pairs* *ij* *,* *i* < *j* *, columns indexed by pairs* *kℓ* *,* *k* < *ℓ* *, and*

$$A_{ij,k\ell }=\left\{\begin{array}{cc} 1\; & \text{ if }i\text{ and }j\text{ are on opposite sides of the split }\sigma_{\left(k\ell \right)}\\ 0\; & \text{ otherwise.} \end{array}\right.$$

The matrix **A** has determinant $\pm 2^{\frac{\left(n-1\right)\left(n-2\right)}{2}}$ and so is non-singular (Bryant and Dress, 2006).

We let **d** and ***λ*** denote vectors of observed distances and split weights, so

$$\begin{array}{ll} d_{ij} & =d\left(x_{i},x_{j}\right)\\ \lambda_{k\ell } & =\lambda_{\sigma_{k\ell }}, \end{array}$$

for all *ij* and *kℓ*.

The NNLS problem to be solved is to minimize

$$f\left(\lambda \right)=\frac{1}{2}\|A\lambda -d{\|}^{2},$$

subject to the constraint that ***λ***_*kℓ*_ ≥ 0 for all *k* < *ℓ*. The function *f* has gradient

$$\nabla f\left(\lambda \right)=A^{T}\left(A\lambda -d\right),$$

and a (non-negatively constrained) optimum given by the unique vector ***λ*** satisfying (∇*f*(***λ***))_*kℓ*_ ≥ 0 for all *kℓ* and (∇*f*(***λ***))_*kℓ*_ = 0 for all *kℓ* such that ***λ***_*kℓ*_ > 0.

#### 4.1.2 Fast matrix multiplication

In practical applications, the matrix **A** can be quite large. For example, if *n* = 1,000, then **A** has approximately 500,000 rows and columns and over 250 billion entries. However, we do not construct **A** explicitly in memory. Instead, we use the matrix implicitly, taking advantage of the structure in the matrix to derive efficient algorithms for computing **Ax**, **A** ^*T*^ **x**, and **A** ^−1^ **x** for a vector **x**. As we shall see, each of these can be computed in *O*(*n* ^2^) time, which is linear in the number of entries of **x**.

1. If **y** = **Ax**, then

$$y_{i\left(i+1\right)}=\overset{i}{\underset{k=1}{\sum }}x_{k\left(i+1\right)}+\overset{n}{\underset{k=i+2}{\sum }}x_{\left(i+1\right)k}\text{ for }i=1,\ldots ,n-1,$$ (3)

$$y_{i\left(i+2\right)}=y_{i\left(i+1\right)}+y_{\left(i+1\right)\left(i+2\right)}-2x_{\left(i+1\right)\left(i+2\right)}\text{ for }i=1,\ldots ,n-2,$$ (4)

$$y_{ij}=y_{i\left(j-1\right)}+y_{\left(i+1\right)j}{-y}_{\left(i+1\right)\left(j-1\right)}-2x_{\left(i+1\right)j}\text{ for }1\leq i<i+3\leq j\leq n.$$ (5)

2. If **y** = **A** ^*T*^ **x**, then

$$y_{i\left(i+1\right)}=\overset{i-1}{\underset{k=1}{\sum }}x_{ki}+\overset{n}{\underset{k=i+1}{\sum }}x_{ik}\text{ for }i=1,\ldots ,n-1,$$ (6)

$$y_{i\left(i+2\right)}=y_{i\left(i+1\right)}+y_{\left(i+1\right)\left(i+2\right)}-2x_{i\left(i+1\right)}\text{ for }i=1,\ldots ,n-2,$$ (7)

$$y_{ij}=y_{i\left(j-1\right)}+y_{\left(i+1\right)j}{-y}_{\left(i+1\right)\left(j-1\right)}-2x_{i\left(j-1\right)}\text{ for }1\leq i\text{ and }i+3\leq j\leq n.$$ (8)

3. If **y** = **A** ^−1^ **x**, then

$$y_{12}=\left(x_{12}+x_{1n}{-x}_{2n}\right)/2,$$ (9)

$$y_{1j}=\left(x_{1j}+x_{\left(j-1\right)n}{-x}_{1\left(j-1\right)}{-x}_{jn}\right)/2\text{ for }2<j<n,$$ (10)

$$y_{1n}=\left(x_{1n}+x_{\left(n-1\right)n}{-x}_{1\left(n-1\right)}\right)/2,$$ (11)

$$y_{i\left(i+1\right)}=\left(x_{i\left(i+1\right)}+x_{\left(i-1\right)i}{-x}_{\left(i-1\right)\left(i+1\right)}\right)/2\text{ for }2\leq i<n,$$ (12)

$$y_{ij}=\left(x_{ij}+x_{\left(i-1\right)\left(j-1\right)}{-x}_{i\left(j-1\right)}{-x}_{\left(i-1\right)j}\right)/2\text{ for }2\leq i\text{ and }i+3\leq j\neq n.,$$ (13)

Eq. 3 is obtained by summing over all splits separating two taxa adjacent in the order, while (6) involves a sum over all *n* − 1 pairs separated by a split {*x*_*i*_}|*X* − {*x*_*i*_}. All other identities are consequences of the observation that if **y** = **Ax**, then

$$\begin{array}{lll} x_{i\left(i+1\right)} & =\left(y_{\left(i-1\right)i}+y_{i(i+1}{-y}_{\left(i-1\right)\left(i+1\right)}\right. & 1<i\leq n-1\\ x_{ij} & =\left(y_{ij}+y_{\left(i-1\right)\left(j-1\right)}{-y}_{i\left(j-1\right)}{-y}_{\left(i-1\right)j}\right)/2 & 1<i<j\leq n. \end{array}$$

This is essentially the combinatorial Crofton formula given by Chepoi and Fichet (1998), though with different indexing.

#### 4.1.3 Numerical error

As the number of taxa increases, the runtime complexity of the algorithms clearly becomes critical. In our experience, the control of numerical errors is of equal, or possibly greater, importance. These issues are not new; there is a vast literature on numerical errors and their impact on least squares problems; for comprehensive introductions, see Dahlquist and Björck (2003) and Golub and Van Loan (2013).

As an illustration, consider the algorithms for computing **Ax** and **A** ^−1^ **x**, outlined in the previous section. Suppose that the number of taxa *n* equals 500, and we simulate an *n* (*n* − 1)/2-dimensional vector **x** by drawing each entry independently from a standard uniform distribution. If we compute **y** = **Ax** and then compute **z** = **A** ^−1^ **y**, then we might expect

$$z=A^{-1}y=A^{-1}Ax=x.$$

In practice, we have found that, on average,

$$\|x-z{\|}_{1}=\underset{ij}{\sum }|x_{ij}{-z}_{ij}|\approx 1.2\times 10^{-7},$$

while

$$\|x-z{\|}_{2}=\sqrt{\underset{ij}{\sum }{\left(x_{ij}{-z}_{ij}\right)}^{2}}\approx 7.3\times 10^{-10}.$$

The exact figure will depend on the choice of the norm, on *n* and also on the architecture of the computer and software. Independent of the details, we should not expect the calculation of gradients, function values, and estimates of split weights to be exact.

By itself, this level of imprecision will not necessarily create problems, as it is hard to envisage a data set where differences to the order of 10^–7^ would have a noticeable impact on the analysis. Unfortunately, the NNLS problem that we have to solve is *ill-conditioned*, that is, small changes in the data or small errors in the computation can get amplified and cause serious difficulties. The *condition number* of a matrix with respect to the standard Euclidean norm ‖ ⋅‖ is defined (Golub and Van Loan, 2013) as

$$\kappa_{2}\left(A\right)=\|A{\|}_{2}\|\|A^{-1}{\|}_{2}=\underset{\|x\|=1}{\max}\|Ax\|\cdot \underset{\|x\|=1}{\max}\|A^{-1}x\|.$$

The larger the condition number of **A**, the more sensitive the solution of the linear equation **Ax** = **y** is to changes in **y**.

We do not have an exact analytical formula for the condition number *κ*_2_(**A**) of *A*, but calculations for *n* ≤ 50 suggest that *κ*_2_(*A*) grows faster than $\frac{4}{5}\frac{n\left(n-1\right)}{2}$ . Hence, as a heuristic, if **y** has an error with magnitude *ϵ*‖**y**‖, then when solving **Ax** = **y** with *n* taxa, we should not expect an error in **x** with magnitude less than 10^5^ *ϵ*|**x**|. This is a property of the NNLS problem, not of the data or of the computer that the calculations are being carried out on.

These numerical issues have practical ramifications. Because of the size of the problems that we consider, we will typically use iterative algorithms to solve the various linear systems which arise. Numerical issues can lead to a failure of convergence for these methods. Even when the methods do converge, we still have to specify some kind of stopping condition. Any stopping condition needs to be realistic with respect to the level of accuracy which could possibly be achieved. Even deciding whether a solution is approximately optimal becomes challenging.

### 4.2 Methods

NNLS is a classical problem of numerical optimization and several strategies are available. We review three separate approaches and describe the modifications that we have developed to adapt them to our problems.

To facilitate comparisons between the methods, we use the same initial split weights and the same criterion for convergence each time. The initial split weights are determined by

$$\lambda =A^{-1}d\;$$

using the aforementioned methods and replacing the negative entries with zeroes. If all entries of **A** ^−1^ **d** are initially non-negative, then this will be the optimal NNLS solution and no iterations are necessary. As a consequence, if **d** is already a circular metric, then split weights are determined optimally in *O*(*n* ^2^) time.

If the initial conditions are not already optimal, the iterative algorithms are called until a convergence criterion is satisfied. For any putative solution ***λ***, we compute the projected gradient **g** defined by

$$g_{ij}=\left\{\begin{array}{cc} {\left(\nabla f\left(\lambda \right)\right)}_{ij}\; & \lambda_{ij}>0;\\ \min\left({\left(\nabla f\left(\lambda \right)\right)}_{ij},0\right)\; & \lambda_{ij}=0. \end{array}\right.$$

Then, ‖**g**‖^2^ = 0 if and only if ***λ*** solves the NNLS problem. To account for numerical imprecision, we consider that the method has converged when ‖**g**‖^2^ < *δ*. The default value that we use for *δ* is 10^–8^‖**A** ^*T*^ **d**‖^2^, which scales with *n* and is similar to the stopping criteria used in the SplitsTree4 method.

#### 4.2.1 Active-set method

The active-set method (Lawson and Hanson, 1995; Nocedal and Wright, 2006) is one of the most widely used algorithms for solving NNLS problems. It is the method used for computing split weights in the SplitsTree4 (Huson and Bryant, 2006). The *active set* is a set of indices $A=\left\{ij:\lambda_{ij}=0\right\}$ for which the corresponding split weight is zero. Given A , we define an *equality-constrained subproblem*

$$\underset{\lambda }{\min}\|A\lambda -d\|,$$

subject to the constraint that ***λ***_*ij*_ = 0 for all ij∈A . Note that this subproblem does not constrain the elements of ***λ*** to be non-negative. The aim is to determine an active set A such that

1. The solution ***λ*** * to the equality-constrained problem is non-negative and
2. $\nabla f{\left(\lambda^{*}\right)}_{ij}\geq 0$ for all ij∈A ,

so that ***λ*** * is the solution to the NNLS problem.

During each iteration, we update the active set A and feasible solution ***λ***, so that ***λ***_*ij*_ = 0 for all ij∈A . The iterations are designed so that ‖**A*λ*** − **d**‖ decreases monotonically.

Algorithm 2 Active-Set Method

1: ***λ*** ← any feasible solution
2: A←∅

3: **loop**
4:  **repeat**
5:   let ***λ*** * minimize ‖**A*λ*** − **d**‖ such that ***λ***_*ij*_ = 0 for all ij∈A

6:   **if** ***λ*** * is infeasible **then**
7:    let ***λ*** be the feasible point on the line from ***λ*** to ***λ*** * which is closest to ***λ*** *
8: $A\leftarrow \left\{ij:\lambda_{ij}=0\right\}$

9:    **end** **if**
10:  **until** ***λ*** * is feasible
11:  **g** ←**A** ^*T*^(**A*λ*** * − **d**)
12:  **if** **g**_*ij*_ ≥ 0 for all ij∈A **then**
13:   **Return** ***λ*** *
14:  **end** **if**
15:  Remove the pair *ij* from A that minimizes **g**_*ij*_
16:  ***λ*** ← ***λ*** *
17: **end** **loop**

A key component of the active-set method is the algorithm used to minimize ‖**A*λ*** − **d**‖ over all ***λ*** such that ***λ***_*ij*_ = 0 for all ij∈A . Let **B** denote the matrix **A** restricted to columns *not* indexed by pairs in A . This equality-constrained minimization is equivalent to finding **y** which solves the normal equation:

$$B^{T}\left(By-d\right)=0.$$

Two standard algorithms for solving this kind of linear system are QR decomposition and Cholesky decomposition (Golub and Van Loan, 2013). Neither is practical for the size of problem that we are dealing with here due to both running time and memory requirements. In SplitstreeCE, we use CGNR (Saad, 2003), a version of the conjugate gradient algorithm designed for solving normal equations. The algorithms of Section 4.1.2 can be used to efficiently multiply vectors by **B** or **B** ^*T*^ without having to construct either matrix in memory.

The classical implementation of the active-set method only allows the active set A to change by one variable, or a few variables, in each iteration (steps 8 and 15). In our experience, the global solution typically has Ω(*n* ^2^) entries in the active set, requiring many iterations just to insert sufficiently many entries in A . For this reason, we allow many variables to enter the active set in each iteration. We choose *ρ* ∈ (0, 1), sort the entries in $\left\{ij:\lambda_{ij}^{*}<0\right\}$ and add a proportion *ρ* of these entries to A , choosing those for which $\frac{\lambda_{ij}}{{\left(\lambda {-\lambda }^{*}\right)}_{ij}}$ is the smallest. We use a default value of *ρ* = 0.6 in SplitsTreeCE.

**FIGURE 2:**
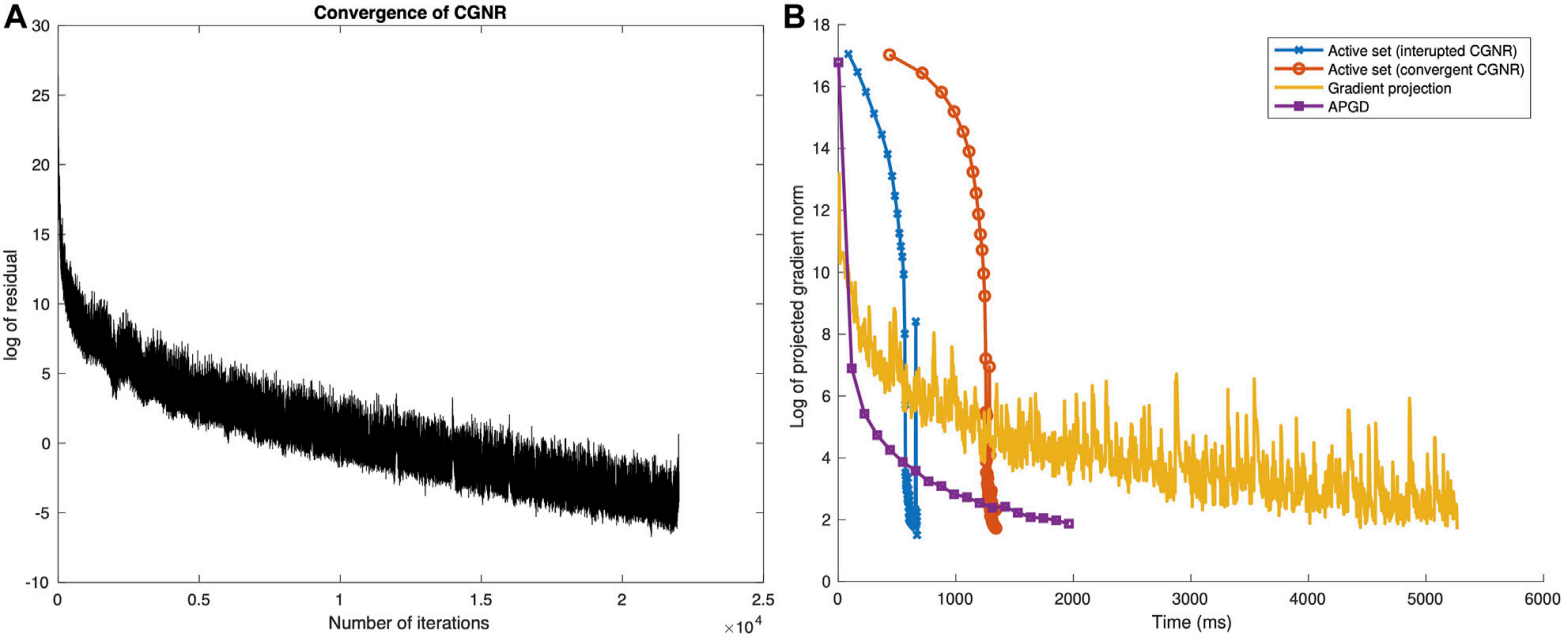
**(A)** Convergence (in the norm of the gradient) of the CGNR method and **(B)** convergence (in the norm of the projected gradient) of the active-set, gradient projection, and APGD methods, as a function of wall-clock time.

Under exact arithmetic, CGNR typically converges to a solution in finite time much more quickly than exact linear equation solvers. In practice, for large problems, numerical problems can cause the method to break down and either converge very slowly or not converge at all. We found that for large problems, the algorithm often took too long to converge. Figure 2A gives a plot of the (log) residual *versus* iteration for a typical call to CGNR on the *Streptococcus agalactiae* data of Morach et al. (2018). The graph shows the initial rapid convergence followed by a long tail of linear convergence. The residual reduces each iteration, but too slowly. We tried implementing periodic restarts, but this had little effect. We also designed a number of preconditioners (Saad, 2003) but were unable to find one which reliably improved the performance.

Our strategy is to not run CGNR to convergence. This is similar to a standard restart, the difference being that we update the active set between runs. We bound the number of iterations of the CGNR by 50 or the number of taxa, whichever was larger.

Figure 2B shows the rate of convergence plot for the active-set method as applied to the *S. agalactiae* data, both with and without running CGNR to convergence, as a function of wall-clock time (on a MacBook Air 2021). For both curves, the error initially remains high, before dropping rapidly. This is to be expected, and reflects the fact that once a good active set is identified, the convergence is extremely rapid. Also note that restricting the number of iterations of CGNR gives a two-fold increase in speed.

#### 4.2.2 Gradient projection method

In each iteration of the active set method, we start at a feasible point ***λ*** and move as far as we can toward the (approximate) solution ***λ*** * of the subproblem, while still remaining feasible. The gradient projection method takes a different approach. Instead of moving along the line from ***λ*** to ***λ*** * and stopping as soon as the point becomes infeasible, it moves along the line from ***λ*** to ***λ*** * and projects any infeasible points back into the feasible region (Nocedal and Wright, 2006).

Given a point ***λ***, let *π*(***λ***) denote the feasible point which is closest to ***λ***, that is, *π*(***λ***) is a vector with

$$\pi {\left(\lambda \right)}_{ij}=\left\{\begin{array}{cc} \lambda_{ij}\; & \text{ if }\lambda_{ij}\geq 0\\ 0\; & \text{ if }\lambda_{ij}<0. \end{array}\right.$$

More compactly, *π*(***λ***)_*ij*_ = max(***λ***_*ij*_, 0). For the gradient projection algorithm, we select a search direction (in this case, the search direction **p** = −∇*f*(***λ***) of the steepest descent) and carry out a one-dimensional line search with respect to the function

$$q\left(t\right)=f\left(\pi \left(\lambda +tp\right)\right)=\frac{1}{2}\|A\left(\pi \left(\lambda +tp\right)\right)-d{\|}^{2}.$$ (15)

Each pair *ij* of indices with **p**_*ij*_ < 0 is associated to a *breakpoint* *t*_*ij*_ such that ${\left(\lambda +t_{ij}p\right)}_{ij}=0$ . When *t* < *t*_*ij*_, we have $\pi {\left(\lambda +t_{ij}p\right)}_{ij}>0$ ; otherwise, $\pi {\left(\lambda +t_{ij}p\right)}_{ij}=0$ . For values of *t* lying between consecutive breakpoints, *q*(*t*) is quadratic.

Nocedal and Wright (2006) propose a line search strategy which examines breakpoints in the order of increasing magnitude, stopping when *q*(*t*) reaches a local minimum. This strategy works well for small numbers of taxa, however, when *n* is large, the number of breakpoints becomes large, the gaps between them become tiny, and the algorithm grinds to a halt.

Instead, we implemented a version of the gradient projection method due to Cartis et al. (2012), though simplified, as we do not require trust regions and do not incorporate regularization. In this approach, the line search can terminate before reaching a local optimum, provided the technical conditions are satisfied (see Conn et al., 2000, Section 12.1; Cartis et al., 2012), conditions which guarantee convergence of the method.

Algorithm 3 Gradient Projection Method

1: ***λ*** ← any feasible solution
2: **while** ***λ*** is not optimal, **do**
3:  **p** ← − **A** ^*T*^(**A*λ*** − **d**)
4:  Let t* be the first local optimum of *q*(*t*) = ‖**A**(*π*(***λ*** + *t* **p**)) − **d**‖
5:  ***λ*** ^*c*^ ← *π*(***λ*** + t*p)
6: $A\leftarrow \left\{ij:\lambda_{ij}^{c}=0\right\}$

7: **end** **while**

We carry out several iterations of the conjugate gradient algorithm to find ***λ*** * such that *f*(***λ*** *) ≤ *f*(***λ*** ^*c*^) and ${\left(\lambda^{*}\right)}_{ij}$ for all ij∈A . We then find *t*, which minimizes

$$f\left(\pi \left(\lambda^{c}+t\left(\lambda^{*}{-\lambda }^{c}\right)\right)\right),$$

and let ***λ*** = *π*(***λ*** ^*c*^ + *t*(***λ*** * − ***λ*** ^*c*^)).

The rate of the convergence plot for the *S. agalactiae* data set is shown in Figure 2B. Initially, the error drops quickly, more quickly than for the active-set method. The rate of convergence then slows, and the projected gradient norm fluctuates significantly from one iteration to the next.

#### 4.2.3 Accelerated projected gradient descent method

The accelerated projected gradient descent (APGD) method (Nesterov, 2003; Mazhar et al., 2015) is a first-order method (using only first derivatives) and yet exhibits a guaranteed rate of convergence. The basis of the method is a projected version of the steepest descent

$$\lambda^{\left(k+1\right)}=\pi \left(\lambda^{\left(k\right)}-\alpha \nabla f\left(\lambda^{\left(k\right)}\right)\right),$$

where (as previously mentioned) *π*(***λ***) is the vector formed by replacing the negative entries of ***λ*** with 0 and *α* is a carefully chosen step length. Nesterov devised several acceleration modifications. These are often described as including “momentum” in the optimization algorithm. We implement an adaptive scheme which resets the momentum term if the objective function increases during an iteration.

Algorithm 4 Accelerated Projected Gradient Descent Method

1: ***λ*** ^(0)^ ← any feasible solution, **y** ^(0)^ ← ***λ*** ^(0)^
2: Choose *θ*_0_ ∈ (0, 1)
3: **for** *k* = 0, 1, 2, … until convergence **do**
4:  **g** ←**A** ^*T*^(**Ay** ^(*k*)^ − **d**))
5: $\lambda^{\left(k+1\right)}\leftarrow \pi \left(y^{\left(k\right)}-\frac{1}{\left\|A^{T}A\right\|}g\right)$

6: $\theta_{k+1}\leftarrow \frac{{-\theta }_{k}^{2}+\theta_{k}\sqrt{\theta_{k}^{2}+4}}{2}$

7: $\beta_{k+1}\leftarrow \frac{\theta_{k}\left(1-\theta_{k}\right)}{\theta_{k}^{2}+\theta_{k}}$

8:  **y** ^(*k*+1)^ ← ***λ*** ^(*k*+1)^ + *β*_*k*+1_(***λ*** ^(*k*+1)^ − ***λ*** ^(*k*)^)
9:  **if** **g** ^*T*^(***λ*** ^(*k*+1)^ − ***λ*** ^(*k*)^) > 0 **then**
10:   **y** ^(*k*+1)^ ← ***λ*** ^(*k*+1)^
11:   *θ*_*k*+1_ ←*θ*_0_
12:  **end** **if**
13: **end** **for**

In our experience, there was essentially no difference in performance for *α*_0_ = 0.1, 0.5, 0.9, or 1.0. The plot in Figure 2B shows a steady rate of convergence, initially fast and then leveling off.

### 4.3 Performance comparison

To compare the different approaches described above, we selected 1,200 prokaryotic genomes that have a mash distance of <0.3 from *Escherichia coli* K12, using a sketch size of 10,000 and k-mer size of 21 (Ondov et al., 2016) and computed all pair-wise mash distances between them. From this distance matrix, we randomly subsampled 20 replicates of smaller distance matrices of sizes *n* = 50, 100, 140, *…*, 1,000. For each such replicate, we computed a circular ordering and then applied the active-set method, gradient projection method, or APGD method, as well as the “SplitsTree4” method that is the implementation of the active-set method that uses SplitsTree4 (Huson and Bryant, 2006).

**FIGURE 3:**
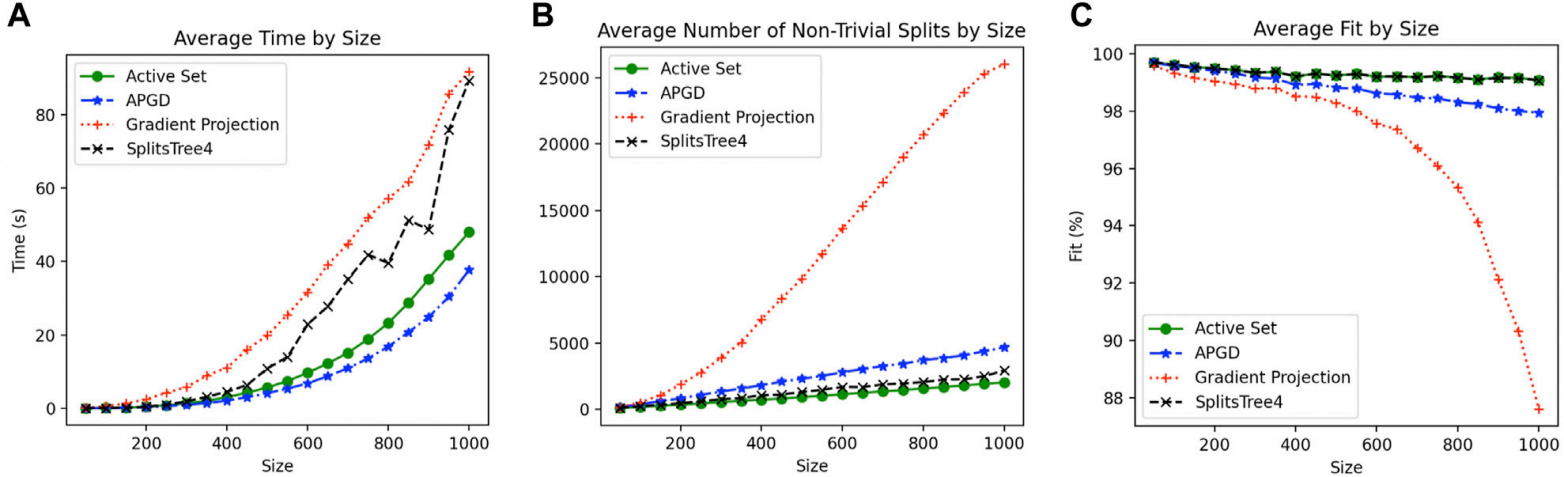
For each data set of size 50–1,000, averaged over 20 replicates per size, and for each of four constrained least-squares approaches, we report **(A)** wall-clock time in seconds, **(B)** number of internal splits, and **(C)** percent fit (as defined in Section 6.2.1).

The results of this study, summarized in Figure 3, suggest that the APGD is the fastest method, while the active-set method is the second fastest, providing the best fit (equal to the fit of the old implementation of the same method in our program SplitsTree4), and the smallest number of splits. The gradient projection method runs the slowest, producing many more splits, with a much poorer fit. Times reported are the wall-clock times, running all four methods in parallel on a Mac Pro 2020 workstation. Based on these observations (which we also confirmed on two other data sets that are not shown here), in our program the SplitsTreeCE, we made the (modified) active-set method the default method.

## 5 Third step: construction of a network

The third main step of the NeighborNet is to construct and draw a network that represents the set of circular splits calculated in the first two parts of the method. This step is described in Dress and Huson (2004) and a simplified visualization is provided in Bagci et al. (2021).

### 5.1 Split networks

As discussed above, if we are given a set of splits S on *X* that is compatible (and contains all trivial splits), then a phylogenetic tree *T* can be used to represent the set of splits; there is a one-to-one correspondence between splits and edges in the tree. In a drawing, the edges are usually scaled to represent the corresponding split weights.

More generally, *any* set of splits S on *X* can be represented by a *split network* *N*. In such a network, each split *S* = *A*|*B* is represented by a set of edges (usually drawn as parallel lines of the same length) with the property that deleting those edges will result in exactly two connected components, one containing the set of taxa *A* and the other containing *B*. The convex hull algorithm (Bandelt et al., 1995; Dress and Huson, 2004) can be used to compute a split network for any set of splits, resulting in an exponential number of nodes and edges in the worst case.

**FIGURE 4:**
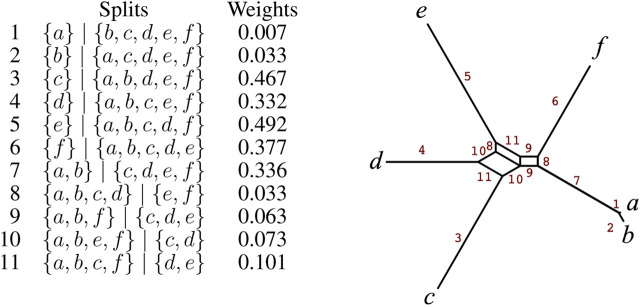
For 11 listed splits on *X* = {*a*, *b*, *c*, *d*, *e*, *f*} and weights, we show their representation as a split network, with edges labeled 1 − 11 to indicate the associated splits.

The first two steps of the NeighborNet compute a circular ordering *θ* = (*x*_1_, *x*_2_, *…*, *x*_*n*_) and a set of splits S that are compatible with that ordering. For these data, there exists a split network *N* that represents S that is outer-labeled planar, that is, it can be drawn in such a way that no two edges cross and all taxa appear on the perimeter of the network. We show an example in Figure 4.

Here, we summarize the main properties of a split network.

(N1) Each edge is associated with a single split, and each split is associated with at least one edge.

(N2) Removing all the edges associated with some split divides the network into two connected components. Each component contains the taxa on one side of the split.

Both of these properties also hold trivially for unrooted phylogenetic trees. They imply that, for any two taxa *x* and *y*, a path from *x* to *y* in the network will cross at least one edge associated to each split that separates *x* and *y*. In fact, a stronger property holds.

(N3) The edges along any geodesic (shortest path) in the graph are associated with different splits.

Hence, for any taxa *x* and *y*, any shortest path from *x* to *y* contains exactly one edge associated with each split separating *x* and *y*. Alternatively, we can replace (N3) by the following convexity property.

(N3’) For any split, the two associated connected components are convex, that is, each contains all the shortest paths between any two nodes.

Properties (N1) to (N3) guarantee that the edges along any shortest path between the taxa correspond exactly to the splits separating those taxa. As a consequence, the total length of the shortest path between the two taxa *x* and *y* is exactly

$$d\left(x,y\right)=\underset{S\in S,S\left(x\right)\neq \bar{S}\left(x\right)}{\sum }\lambda \left(S\right),$$

where the sum is over all splits that separate *x* and *y*. This implies that the split network is a faithful representation of the decomposition into split metrics.

Split networks are known in other branches of mathematics as *partial cubes*, which mean that there is a map from the graph to a hypercube that preserves distances. It follows from this that we can assume the following property for any drawing of a split network.

(N4) The edges associated with a split *A*|*B* are parallel line segments with the length equal to the weight of the split *A*|*B*.

### 5.2 Planar split networks

To draw a split network, we have to assign coordinates to all nodes. We will discuss this for circular splits. The NeighborNet is an attractive visualization technique because of the following result.

Theorem 5 *A set of splits* S *on* *X* *that is compatible with a circular ordering* *θ* = (*x*_1_, *…*, *x*_*n*_) *can be represented by a split network* *N* *that is outer-labeled planar.*

**FIGURE 5:**
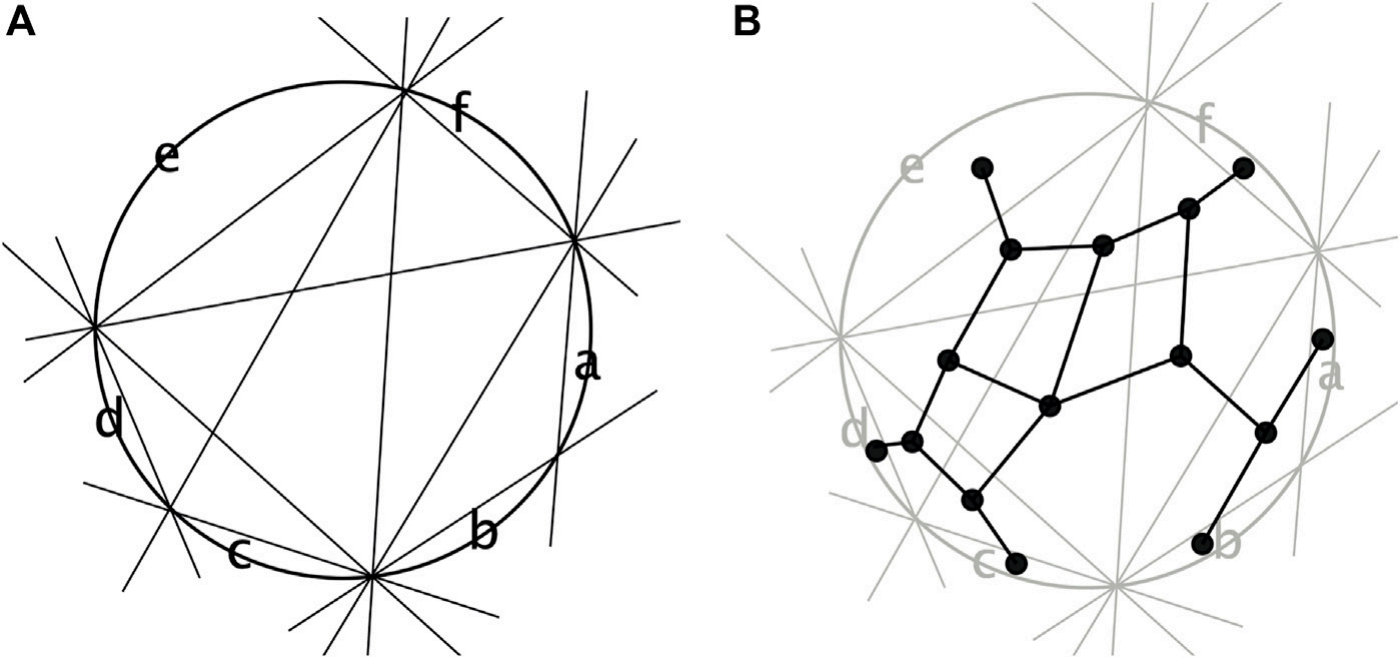
**(A)** Splits from Figure 4 drawn as lines separating points on a circle and **(B)** the corresponding dual graph.

One way to show this is using de Bruijn’s dualization (de Bruijn, 1986). We place the taxa on the unit circular in the order *θ* and then represent each split *A*∣*B* by a line that separates those two parts of the split. This is known as a line arrangement. The dual is the graph *N* obtained by placing a node on each taxon and in each bounded region of the arrangement. The edges are placed between any two nodes whose regions intersect along a line segment. This construction is demonstrated in Figure 5.

A general characterization of when a collection of splits S has a planar splits network was worked out by Balvociute et al. (2017) using oriented matroids and the Bohne–Dress theorem (Bohne et al., 1992). The result of which was more general than we require since the NeighborNet only produces networks from circular splits. The first proof that these split networks have planar drawings was given by Dress and Huson (2004), who also provide the equal-angle algorithm for efficiently constructing and drawing these networks (also see Gambette and Huson, 2008; Phipps and Bereg, 2011). The equal-angle algorithm is the one usually used to perform step 3 of the NeighborNet algorithm, as implemented in our SplitsTree programs (Huson and Bryant, 2006).

In both the equal-angle algorithm and outline algorithm (Bagci et al., 2021), we first assign an angle to each taxon based on its position in the ordering *θ* = (*x*_1_, *…*, *x*_*n*_), setting $\alpha \left(x_{i}\right)=\frac{\left(i-1\right)}{n}360{}^{\circ}$ . For each split *S*, we define its angle *α*(*S*) as the average angle assigned to the taxa contained in $\bar{S}\left(x_{1}\right)$ . The edges representing *S* are drawn using this angle and their lengths reflect the weight of the split (using additional considerations to place the edges; for more details, see the cited works).

## 6 Interpretation of NeighborNet output

Split networks produced using the NeighborNet are a generalization of phylogenetic trees and must be interpreted accordingly (see Figure 1). In this section, we give some general guidelines to help this process.

### 6.1 The networks do not explicitly depict evolutionary scenarios

The most important fact to take into account is that a split network does not provide an *explicit* evolutionary scenario (Huson et al., 2010); internal nodes usually do not correspond to putative ancestors and edges do not always represent different lineages or reticulation events. Such a network provides an *implicit* representation of evolution in which the key features are the splits and their weights (or lengths).

### 6.2 The networks represent distances

In a phylogenetic tree, the length of the path between two taxa represents the inferred evolutionary (patristic) distance between the two taxa. Distance-based methods typically work by first estimating pair-wise distances between sequences and then finding an evolutionary tree so that the distances in the tree approximate the distances used as input.

In a split network, the length of the *shortest* path between two taxa represents the inferred evolutionary (patristic) distance. Because split networks are a generalization of phylogenetic trees, that often allow a better approximation of the input distances than can be realized using a tree.

In SplitsTree, the closeness of approximation is measured using a fit statistic. Let *d*_*ij*_ denote the input distances and *p*_*ij*_ denote the distances in the network. The fit statistic is defined as

$$\text{fit}=1.0-\frac{\sum_{i,j}{\left(d_{ij}-p_{ij}\right)}^{2}}{\sum_{i,j}d_{ij}^{2}},$$

reported as a percentage. If the network distances exactly match the input distances, then the fit is 100%.

In our experience, the fit statistic for biological sequence data is usually above 90%, indicating that the distances in the network provide a good approximation of the input distances. However, it is easy to construct the input data that give rise to a poor fit. In particular, Euclidean distances are not well suited as input, although they fit well with multi-dimensional scaling techniques. A low-fit statistic indicates that the network provides only a poor approximation of the input distances and so inferences should be made from the network with caution.

### 6.3 The networks are not based on generative model

A widespread trend in statistical phylogenetics is to carry out inference using complex stochastic and generative models. The models are constructed so as to mimic as many different evolutionary processes as possible. These analyses prioritize statistical power, which makes good sense if the model is reasonable and there are no surprises in the data.

The NeighborNet algorithm is not based on a generative model. There are no model parameters or prior distributions controlling where and how frequently reticulations occur. The only model of data variability is the assumption of noise in the distance data implicit in the least squares approach.

Because the method is statistically consistent on circular metrics, we can say something about what the NeighborNet will do when applied to data generated according to a corresponding phylogenetic model. When the input distances are additive, the NeighborNet will return the corresponding tree. When the distances are almost additive, the NeighborNet will return a split network that is close to a tree. If a distance is generated from a mixture of trees and the combined splits of those trees are circular, then the NeighborNet will represent the averaged splits of the trees. This applies, for example, to a pair of trees which differ by a single subtree transfer operation.

It is possible to bind the expected error that is introduced by applying distance corrections for alignments composed of multiple heterogeneous blocks (Bryant et al., 2003).

### 6.4 The networks are akin to phylogenetic scatter plots

A useful analogy to use when interpreting the output of the NeighborNet is a scatter plot. Suppose that we have a collection of pairs of values (*x*_*i*_, *y*_*i*_), with 1 ≤ *i* ≤ *n*, and assume that we suspect that the values are generated using a simple model

$$y_{i}=\alpha x_{i}+\beta +\epsilon_{i},$$

where the two variables *α* and *β* are the parameters being inferred and the *ϵ*_*i*_ values are independent random variables. The true model is essentially a line and so a model-based inference would focus on the line inferred or the corresponding parameters, perhaps with their uncertainties. When we produce the scatter plot, we are not making assumptions about the underlying model, nor are we necessarily making concrete progress toward inferring the true values of the parameters. Instead, we are learning more about the *data* and their suitability for the model-based analysis that we might have planned.

Just as a scatter plot might reveal outliers or strange patterns in the data, a NeighborNet might reveal errors in sequencing or labeling, or perhaps indicate the potential of conflicting signals in the data which might make use wary of assuming the suitability of an analysis based on a single tree.

In this sense, the process of going from distance data to a split network in the NeighborNet is closer to a data transform than a model-based inference. The method shares similarities to Hadamard conjugation (Hendy and Penny, 1993), which also produces a set of splits with weights. In the case that the splits are circular, the NeighborNet applied to correct distances provides an approximation of the spectrum produced by Hadamard conjugation; an approximation which gets more and more accurate as the sequence length increases.

## 7 Open problems and related work

Despite 20 years of work examining and improving the NeighborNet, there are several problems that remain open.

### 7.1 Simplifying the NeighborNet ordering algorithm

At present, the NeighborNet algorithm uses a two-stage selection process when choosing how to join chains: first the two chains are chosen and next the ends to be joined are selected for either chain. We wish to determine whether this step can be reduced to a single-stage criterion or whether such a simplified algorithm is impossible is determined, see Bryant et al. (2007).

### 7.2 Searching through circular orderings

A standard strategy for inferring a phylogeny is to start with a tree that is determined using a heuristic such as the neighbor-joining and then carry out local search to optimize some criteria. The same strategy could be implemented for inferring circular networks; however, the question is which criterion to use.

Bandelt and Dress (1992b) observed that if *d* is a circular metric, then a permutation *σ* corresponds to a circular ordering (*x*_*σ*(1)_, *x*_*σ*(2)_, …, *x*_*σ*(*n*)_) compatible with *d* if and only if the tour length

$$\ell \left(\sigma \right)=d\left(x_{\sigma \left(1\right)},x_{\sigma \left(2\right)}\right)+d\left(x_{\sigma \left(2\right)},x_{\sigma \left(3\right)}\right)+\cdots +d\left(x_{\sigma \left(n-1\right)},x_{\sigma \left(n\right)}\right)+d\left(x_{\sigma \left(n\right)},x_{\sigma \left(1\right)}\right),$$

is minimal. Hence, we can infer a circular split network consistently by solving the travelling salesman problem (TSP). This approach was explored by Eslahchi et al. (2010), who proposed a simple insertion scheme followed by randomized local search to find an ordering with small total length.

One problem in using the TSP to infer the ordering is that it appears highly vulnerable to noise in the distance estimates. With such a large number of different pairs, there is a reasonable chance that one distance might be substantially underestimated, with significant, random, impact on the minimal tour.

A potential solution for this problem is to use a related criterion which involves averages of larger numbers of distances, and thereby reduces the impact of the outliers. Suppose that *X* is the set of taxa and *Y* ⊆ *X*. The restriction *d*|_*Y*_ of the distance matrix to elements in *Y* will also be a circular metric. Furthermore, if *σ* corresponds to a circular ordering compatible with *d*, then the restriction *σ*|_*Y*_ of *σ* to elements in *Y* will be a circular ordering compatible with *d*|_*Y*_. This suggests a criterion

$$\ell_{w}\left(\sigma \right)=\underset{Y\subseteq X}{\sum }w_{\left|Y\right|}\ell \left(\sigma {|}_{Y}\right),$$

where *w* is a set of non-negative weights such that *w*_|*X*|_ > 0. Then, *σ* is compatible with a circular metric *d* if and only if *ℓ*_*w*_(*σ*) is minimized. This criterion is consistent, involves averages of many estimates and can be computed efficiently by carefully determining the contribution of each distance pair *d*(*x*_*σ*(*i*)_, *x*_*σ*(*j*)_).

### 7.3 Faster estimation of split weights

Say that practical implementations of the NeighborNet can take a prohibitive amount of time to run on a data set of several thousand taxa. Then, the computational bottleneck lies in the NNLS estimation of the split weights. The experimental results that we report represent only a small fraction of the total strategies attempted to make the NNLS algorithm run more quickly. As mentioned, the running time is only one factor, and the increase in numerical errors with more taxa is of comparable significance.

In the past (SplitsTree4), we used the active-set method; now, we use an improved implementation of such a method (SplitsTreeCE). Both implementations make repeated calls to CGNR, the conjugate gradient algorithm, so speeding up CGNR would make a direct and substantial impact on the running time of our implementation of the NeighborNet. The standard technique for making conjugate gradients run better is to use preconditioning (Saad, 2003); however, we have been unable to design a preconditioner that gives a reliable improvement in running time. Such a preconditioner would have to take advantage of the special structure of the matrix **A**, restricted to a subset of columns.

It may make more sense to avoid NNLS completely. The least squares method is familiar and mathematically attractive, but does not best capture the error in the observed distances. It may be possible to adopt a different criterion that retains some of the regularization ability of NNLS but can be computed far more efficiently.

### 7.4 NeighborNet for non-distance data

Usually, reducing a data set down to a distance matrix entails a significant loss in information. There have been several investigations into adapting NeighborNet for other types of data.

A *quartet* is an unrooted, binary (fully resolved) phylogeny on four taxa. The quartet with taxa *a* and *b* separated from taxa *c* and *d* by the internal branch is denoted by *ab*|*cd*. One persistent paradigm for constructing phylogenetic trees is to first infer a collection of quartets on different subsets of taxa and use combinatorial algorithms to assemble these quartets into a full phylogeny.

The problem of constructing circular split networks adapts well to quartet data. Grünewald et al. (2007) explored this approach extensively, resulting in the QNet, a method that can be described as a quartet version of the NeighborNet. Hassanzadeh et al. (2012) implemented a simulated annealing algorithm to maximize a quartet-based criterion for circular “super”-networks.

### 7.5 Inferring circular networks from trees

As previously mentioned, we considered different coefficients for updating the distance matrices during the agglomeration step. There is also scope for different weights when computing the distances between clusters in the first selection step. Levy and Pachter (2011) explored the effect of these weights and demonstrated that the weights can be chosen such that the neighbor-joining tree is embedded in the network. A consequence (which also follows from Theorem 2) is that the neighbor-joining algorithm could, by itself, be used to help construct circular split networks (Guo and Grünewald, 2023).

The theorem suggests a new approach to infer split networks:

1. Infer an unrooted phylogenetic tree *T* (e.g., using the neighbor-joining).
2. Search through circular orderings which are compatible with the splits of *T*.
3. Estimate split weights for the corresponding circular splits.

Guo and Grünewald (2023) propose an integer linear programming algorithm for the second step. The PQ-tree–based algorithm for the TSP of Burkard et al. (2005) could also be used, though it might be worth adapting the algorithm to optimize a criterion that is not so vulnerable to random error.

### 7.6 Taking advantage of structure in the alignment

One of the strengths of the NeighborNet is that it only requires distance data, so it can be applied in a wide variety of contexts. This is also one of its weaknesses. The reason is that the process of computing distances from an alignment discards all of the structural information on which groups of sites support which phylogenetic signals.

As an illustration, consider the phi test (Bruen et al., 2006) for recombination, a method which performs well in many situations. The phi test evaluates a statistic that measures the extent to which nearby sites are more compatible than distant sites and tests for recombination by seeing how this statistic compares to those for the same alignment with sites randomly permuted.

In a NeighborNet analysis, permuting the sites has no effect on the distance estimation, so all of the signals in the data that the phi test uses to detect recombination is ignored by the NeighborNet. Addressing this while still maintaining the speed and practicality of the NeighborNet would represent a significant step forward.

## 8 Summary

The NeighborNet algorithm is related to the split decomposition (Bandelt and Dress, 1992a), neighbor-joining, and pyramidal clustering methods (Diday, 1984), yet differs substantially from all these methods. The algorithm is widely used in many different fields due to its ability to quickly visualize data and incompatibilities.

The second step of the program is computationally intensive. This has been a significant practical limitation, one which was quite challenging to overcome. There is also scope for exploring facets of the data which are not preserved in distance data.

The kind of analysis carried out using the NeighborNet is complementary to many of the accepted approaches to phylogenetic, phylogenomic, and phylodynamic analyses. The analysis more resembles a signal transform or spectral analysis than an estimation of model parameters. While the NeighborNet does not address the problem of inferring the finer parameters of a sophisticated model, it is widely used for data representation and exploration.

## Data Availability

The original contributions presented in the study are included in the article/supplementary material; further inquiries can be directed to the corresponding author.
